# Effect of Fixative Solutions in Long-Term Bone Preservation

**DOI:** 10.3390/biology15070567

**Published:** 2026-04-02

**Authors:** Victoria Gulimova, Yuri Krivonosov, Inna Bukreeva, Alexey Buzmakov, Olga Junemann, Victor Asadchikov, Sergey Saveliev

**Affiliations:** 1 Laboratory of Nervous System Development, Avtsyn Research Institute of Human Morphology of Federal State Budgetary Scientific Institution “Petrovsky National Research Centre of Surgery”, 117418 Moscow, Russia; gulimova@yandex.ru (V.G.); junemann@outlook.com (O.J.); embrains@mail.ru (S.S.); 2 National Research Centre “Kurchatov Institute”, 123182 Moscow, Russia; yuri.s.krivonosov@yandex.ru (Y.K.); asad@crys.ras.ru (V.A.); 3 Institute of Nanotechnology, CNR (Consiglio Nazionale delle Ricerche), 00185 Rome, Italy; inna.bukreeva@cnr.it

**Keywords:** 70% ethanol, 10% formalin, fixative solution, Mongolian gerbil humerus, thick-toed gecko digit, micro-CT, bone parameters

## Abstract

Preservation of biological specimens over extended periods is essential for morphological research, especially in contexts where sample collection is limited or unrepeatable, such as spaceflight studies. However, the impact of long-term exposure to chemical fixatives remains underexplored. In this study, we used high-resolution X-ray microtomography and X-ray phase contrast tomography to investigate the stability of mineralized tissues subjected to prolonged chemical fixation. We examined the forelimb digits of geckos (*Chondrodactylus turneri*) preserved for over six years using a protocol involving formalin and ethanol, as well as the humeral bone of gerbils (*Meriones unguiculatus*) stored in fixative for more than four years. The gecko samples originated from the delayed vivarium control group of the Bion-M No. 1 space mission, offering a valuable chance to evaluate the preservation effects on specimens of significant scientific value. Comparative analysis revealed distinct changes in bone volume and linear attenuation coefficient of bone associated with formalin storage, highlighting the need for optimized storage strategies in long-term biorepositories. These findings offer valuable guidance for maintaining morphological fidelity in chemically preserved skeletal tissues.

## Simple Summary

Long-term preservation of biological samples is essential for research, particularly for rare specimens from space missions or unique studies. However, the effects of common preservation solutions on these samples over time remain poorly understood. This study examined bone tissues from geckos and gerbils stored for four to six years in two different preservation solutions: formalin and ethanol. Advanced X-ray imaging technology was used to measure changes in bone size and X-ray linear attenuation coefficient during storage. Bones stored in formalin gradually swelled and lost mineral content, with compact bone tissue showing volume increases of nearly nine % and X-ray linear attenuation coefficient decreases of over five %. In contrast, bones stored in ethanol remained stable with virtually no changes throughout the storage period. These findings help identify the best preservation method for valuable specimens, ensuring accurate and reliable research results. This knowledge is particularly important for irreplaceable samples from space experiments, medical studies, museum collections, and forensic investigations, ultimately improving the quality of scientific research dependent on preserved biological materials.

## 1. Introduction

Chemical fixation is a fundamental method of morphological preservation in biological research. Despite its widespread use, the long-term effects of common fixatives, particularly formalin and ethanol, remain poorly characterized. In this study, we used high-resolution X-ray microtomography (micro-CT) and X-ray phase contrast tomography (XPCT) to evaluate the impact of long-term chemical preservation on mineralized tissues. We focused our analysis on specimens from the thick-toed geckos (*Chondrodactylus turneri*) and the Mongolian gerbils (*Meriones unguiculatus*), including materials obtained from the delayed vivarium control (DVC) group of the Bion-M No. 1 biosatellite mission. Specifically, we examined the effects of a combined preservation protocol involving 35 days in formalin followed by 2309 days in 70% ethanol on the third digits of left forelimb of thick-toed gecko. We also evaluated changes in humeral bone in Mongolian gerbil after over 1565 days of exposure to either 10% buffered formalin or 70% ethanol. These analyses aim to quantify preservation-induced alterations and inform best practices for the long-term storage of biological specimens.

This study builds on our previous research in which analogous specimens (Mongolian gerbil humeral bones stored in 10% neutral buffered formalin) were subjected to micro-CT analysis following a 12-day spaceflight experiment [1]. Research in this area is particularly valuable given the rarity and the importance of biomaterials from space missions, as extensive analysis is not always possible immediately post-flight. Furthermore, logistical constraints in various research contexts often require extended storage of samples between euthanasia of animals and subsequent analysis. This study addresses the critical need for effective long-term preservation methods to maintain the integrity of these valuable samples and ensure their utility for future scientific investigations.

Our research focuses on the two principal methods (formalin and ethanol fixation) used for long-term sample preservation in space-related research. Formalin fixation, a widely used preservation technique, has shown various effects on bone mechanical properties. Some studies have observed changes in bone biomechanics and composition due to hydroxyapatite dissolution. Currey et al. reported a significant decrease in impact strength of bovine cortical bone even after the less than 3 h fixation in 10% formalin (4% formaldehyde) [2]. Other authors revealed the differences in plastic energy absorption between formalin-ethanol mixture (−41%) and ethanol (+37%) after 14 days preservation of sheep’s metacarpal bones compared to fresh specimens. Formalin fixation caused brittleness of cortical bone specimens, while ethanol increased the plastic energy absorption capacity [3]. Nazarian with co-authors showed that 14-days fixation in acid 10% formalin does not alter the elastic mechanical properties of murine femurs and vertebrae but weakened their viscoelastic properties by reducing their ability to dissipate viscous energy [4]. Kikugawa and Asaka found that after storing bovine femur specimens in acidic 10% formalin for 7 to 140 days, the bending properties of the bones improved, while their fracture toughness reduced compared to the control as the preservation time increased. They considered that the reason for the reduction in bone strength was the leaching of Ca and P into the fixative under the action of formic acid in acidic formalin [5]. However, later the same authors [6] showed that during long-term storage, bone minerals are leached into the fixative in both acidic and neutral buffered formalin. Fonseca et al. concluded that the radiographic optical density of bone specimens stored in 10% formalin diminishes with time (0–90 days), irrespective of buffering, which suggests the occurrence of bone demineralization [7]. Zhang et al. found that changes in the mechanical properties of bone during long-term (4–8 weeks) storage in acidic formalin may depend not only on the duration and temperature of storage, but also on the orientation of osteons in the specimen [8].

At the same time, Mick et al. found no significant differences for ultimate bending strength after the mixed formalin with ethanol or 96% ethanol 14-days fixation of human cortical bone specimens, as compared to unfixed-fresh bone specimens [9]. Also, recent research by Feichtinger et al. using micro-CT demonstrated that 24 h fixation in 4% buffered formaldehyde induced minimal, statistically non-significant changes [10]. On the other hand, it was revealed that regardless of buffering, optical density of bone specimens during 10% formalin storage at time zero was significantly higher than that at 15 days, 30 days and 90 days. However, while optical density at 1 day was significantly higher than that at 30 days and 90 days, it did not differ from that at 15 days. There was also no significant difference in density between 30 days and 90 days [7].

It should be noted that the radiographic optical density, like the X-ray linear attenuation coefficient measured in the present study, reflects the mineral content of bone tissue. However, while optical density is a semi-quantitative, film/sensor-based radiographic measure, whereas the linear attenuation coefficient obtained from micro-CT is a fully quantitative, spatially resolved parameter.

As a result, the variety of experimental conditions and approaches has led to inconsistent and sometimes conflicting results in the available studies, making direct comparisons and conclusive interpretations problematic [9]. This fact highlights the complexity of prolonged bone preservation process and emphasizes the critical need for standardized protocols in biomechanical studies, particularly in space-based research where sample integrity is crucial for accurate analysis of microgravity effects on bone tissue.

Ethanol, another common chemical fixative, has also been studied for its effects on bone mechanical properties. Evans (1973) reported that short-term ethanol preservation increased the tensile elastic modulus and strength of cortical bone [11], while Linde et al. found minimal impact on cancellous bone compressive stiffness after long-term ethanol fixation [12].

Our study employs micro-CT imaging to assess fixative-induced changes in the humeral bone of Mongolian gerbil. We examined alterations in X-ray linear attenuation coefficient and volumetric parameters of bone samples exposed to formalin and ethanol fixation during a 1565-day period. We have observed that formalin fixation increased volume morphometric parameters of cortical and trabecular bone while decreasing the linear attenuation coefficient of cortical bone tissue particularly in the diaphysis. Ethanol fixation, on the other hand, showed no changes in morphometric parameters exceeding the experimental spread across the basic regions of the humerus.

X-ray phase contrast tomography of the 4th phalanx in the forelimb of the thick-toed gecko revealed that the bone volume fraction (BV/TV) in the sample, preserved in 10% buffered formalin for 2344 days, was 5.3% higher compared to the sample, which was stored for 2309 days in 70% ethanol following an initial 35-day fixation in 10% formalin.

This research addresses a critical issue in biomedical methodology and has the potential to improve the accuracy of studies that rely on preservation of bone samples. By comparing preservation methods, we aim to establish best practices for the long-term storage of bone specimens, ensuring validity and reproducibility in fields ranging from space biology to the preservation of museum collections and forensic science.

## 2. Materials and Methods

### 2.1. Samples and Fixative Solutions

Specimens included the third digit of the left forelimb of thick-toed gecko (*Chondrodactylus turneri*, Gray, 1864) collected from adult, intact female geckos in the delayed vivarium control group after a 30-day orbital mission aboard the Bion-M No. 1 biosatellite (2013). The delayed vivarium control experiment was conducted from 26 July to 27 August 2013. At the end of the experiment, geckos were euthanised by intraperitoneal injection of Nembutal (200 mg/kg) and their length and weight measured. The third left forelimb digit of two geckos was then separated from the body and preserved in 10% neutral buffered formalin (Richard-Allan Scientific, Kalamazoo, MI, USA, pH 7.4) for 35 days. After this period, the digit of gecko N°21, with a body weight of 18.9 g, was transferred to 70% ethanol, while the digit of gecko N°18 with a body weight of 14.3 g, remained in the formalin solution until 27 January 2020. Both digits were then washed to remove fixative, dehydrated and embedded in paraffin for subsequent examination using XPCT. Thus, from the beginning of fixation to paraffin embedding, sample N°18 was continuously stored in a 10% formalin solution for about 6 years and 5 months (2344 days), while sample N°21 was initially stored in formalin for 35 days, followed by about 6 years, 4 months (2309 days) in 70% ethanol (pH 4.5, measured by Pehanon pH indicator paper 904 24 (Macherey-Nagel, Düren, Germany) with resolution ±0.5 pH units). The storage temperature for both samples throughout the experiment was maintained at 5 °C.

Another specimen was the right and left humerus of an adult male Mongolian gerbil (*Meriones unguiculatus*, Milne-Edwards, 1867). On 13 May 2020, the animal was euthanized by cervical dislocation in accordance with established protocols [13]. After euthanasia, the humeri were immediately removed, separated from soft tissue and subjected to weight and length measurements. The right humerus was 17.0 mm long and weighed 0.09 g, while the left humerus was 17.0 mm long and weighed 0.08 g. The right humerus was then immersed in 10% buffered formalin and the left in 70% ethanol. The specimens were kept in these fixative solutions at an ambient temperature of 22–24 °C for 4 years, 3 months, and 14 days (1565 days) throughout the experimental period.

The pH of the fixatives in containers with gerbil humerus samples was measured using Pehanon pH indicator paper (904 19 with resolution ±0.2–0.25 pH units) on the day of sample fixation (the first day of the experiment) and after 1565 days (at the end of the experiment). Also, at the end of the experiment, the pH of each fixative was measured in a similar container without a sample, which was stored under the same conditions as the container with the sample. At the end of the experiment, the samples were dehydrated in ethanol of increasing concentration and embedded in paraffin for micro-CT examination.

### 2.2. Synchrotron X-Ray Phase Contrast Tomography

Propagation-based XPCT of the gecko digits was performed at the Diamond Manchester Imaging Branchline I13-2 at the Diamond Light Source synchrotron, Oxfordshire, UK [14,15]. A filtered polychromatic “pink” beam (8–30 keV) of parallel geometry was generated by an undulator with a 5 mm gap. For each tomogram, 4001 projection images were acquired in the angle range of 180 degrees of continuous rotation. Images were collected using a pco.edge 5.5 Camera Link detector (PCO AG, Kelheim, Germany) mounted on a visible light microscope of variable magnification. A 4× objective, coupled to a 500 μm LuAG:Ce scintillator, mounted ahead of a 2× lens, provided a total 8× magnification and an effective pixel size of 0.8125 μm. The propagation distance between the sample and the registration system was 390 mm.

### 2.3. X-Ray Micro-CT

The morphometric parameters of the right and left gerbil humerus were studied via micro-CT. The experiment was carried out over 1565 days, from 14 May 2020 to 26 August 2024. The first three tests were performed with an interval of 25–35 days, followed by two tests at 3.5—months intervals. After that, the measurements were taken every 6 months until the end of the experiment. Tomographic measurements of the humerus were performed using the laboratory micro-CT setup “THOMAS” [16] at the NRC “Kurchatov Institute”. The X-rays were generated by an X-ray tube with a molybdenum anode. The characteristic line E = 17.5 keV was isolated by pyrolytic graphite crystal monochromator. For each tomographic scan, 1000 projections were collected in 0.2° increments using a CCD detector (XIMEA xiRay11, Münster, Germany) with a pixel size of 9 × 9 μm^2^. In particular, the characteristic radiation allowed accurate reconstruction of the linear attenuation coefficient, a critical parameter for humeral bone tissue analysis.

### 2.4. Virtual Segmentation

To compute the morphometric parameters of gecko digits, bone tissue virtual segmentation was executed using XPCT images. The segmentation process employed the interactive machine learning software “ilastik version 1.4.0.post1” [17], specifically utilizing the “pixel classification” mode, which implements the “Random Forests” method as a classifier [18].

Virtual segmentation of the humerus bone of Mongolian gerbil was performed by threshold binarization using the Otsu method [19]. A common threshold was used for bone segmentation in all tomographic experiments. Mathematical morphological operations (dilation, erosion, closure, etc.) were applied to segment the cortical and trabecular bone tissue. The boundaries between the basic regions of the humerus, namely the proximal epiphysis and metaphysis (EM) zone, the diaphysis and the distal EM zone, were delineated according to the method described in the paper [1].

## 3. Results

**Figure 1:**
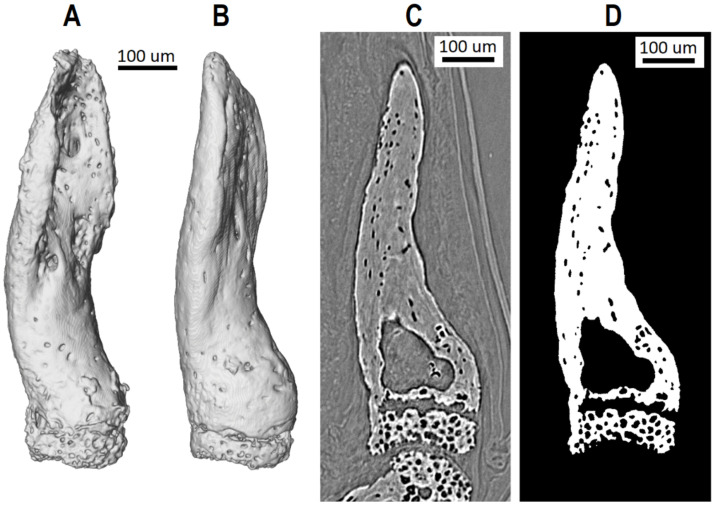
X-ray phase-contrast tomography of the 4th phalanges of gecko’s digits. (**A**,**B**)—the three-dimensional images of samples N°21 and N°18; (**C**)—the longitudinal tomographic sections (N°18); (**D**)—the segmented bone tissue (N°18).

To characterize the bone tissues of the gecko digit phalanges, the following morphometric parameters were assessed: length, bone volume (BV), total volume (TV), and bone volume fraction (BV/TV). Three-dimensional images of gecko’s N°21 and N°18 fingers 4th phalanges are shown in Figure 1A,B. The corresponding longitudinal tomographic sections and the segmented bone tissue are shown in Figure 1C,D. The values of the calculated parameters are presented in Table 1. The analysis reveals that the 4th phalanges of the digits N°18 and N°21 were almost identical in length (N°18 was −3.8% shorter than N°21), but BV, TV and BV/TV were larger for the N°18 specimen stored in 10% formalin (+17.5%, +11.4% and +5.3%, respectively) compared to N°21, stored in 70% ethanol for 2309 days after 35 days in 10% formalin.

The pH of the fixatives in the containers with samples, measured on the day of sample fixation (the first day of the experiment) and after 1565 days (at the end of the experiment) were practically the same: 7.4 and 7.2 for formalin, respectively, and 4.5 and 4.5 for ethanol, respectively. Also, at the end of the experiment, no differences in pH values were found between the container with the sample and without the sample for each fixative.

To assess the alterations in bone tissue of Mongolian gerbil humeral bone resulting from prolonged exposure to fixatives, the following morphometric parameters were analyzed using micro-CT data: TV (mm^3^)—volume of the area of interest (whole sample or humerus basic regions), BV (mm^3^)—volume of bone tissue in the humerus (cortical and trabecular together), Ct.BV (mm^3^)—volume of cortical bone tissue, Tb.BV (mm^3^)—volume of trabecular bone tissue, μ.B (mm^−1^)—linear attenuation coefficient of bone tissue (cortical and trabecular together), μ.Ct (mm^−1^)—linear attenuation coefficient of cortical bone tissue, μ.Tb (mm^−1^)—linear attenuation coefficient of trabecular bone tissue. Linear attenuation coefficients were calculated as median values of tomographic voxels segmented as bone tissue. The trabecular bone volume (Tb.BV) as well as the linear attenuation coefficient (µ.Tb) in the diaphysis of the humerus was not calculated due to the low content of trabeculae in this region (about 1–2% relative to the volume of the entire trabecular bone). The results of the morphometric parameter calculations for both whole samples and the key regions of the humerus at the beginning and end of the experiment, along with the percentage changes in these parameters throughout the study, are presented in Table 2.

**Figure 2:**
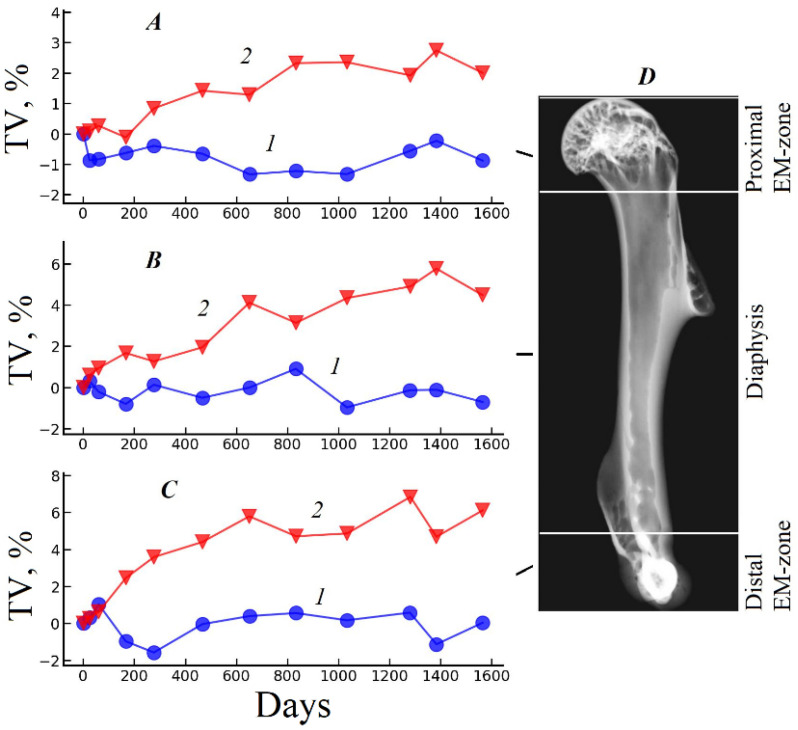
Graphs of changes in the TV of the sample (**A**–**C**) relative to the start of the experiment in the main regions of the humerus (**D**). The results are expressed as percentages. Curves 1—the sample fixed in 70% ethanol; 2—the sample fixed in 10% formalin.

**Figure 3:**
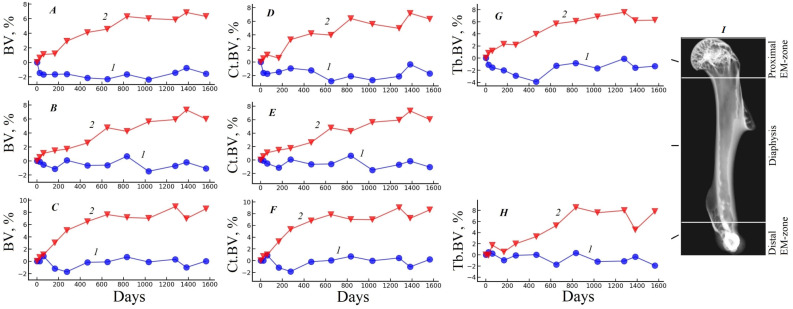
Graphs of changes in bone volume BV (**A**–**C**); cortical bone volume Ct.BV (**D**–**F**); volume of trabecular bone tissue Tb.BV (**G**,**H**) relative to the beginning of the experiment in the main regions of the humerus (**I**). The results are expressed as percentages. Curves 1—the sample fixed in 70% ethanol; 2—the sample fixed in 10% formalin.

**Figure 4:**
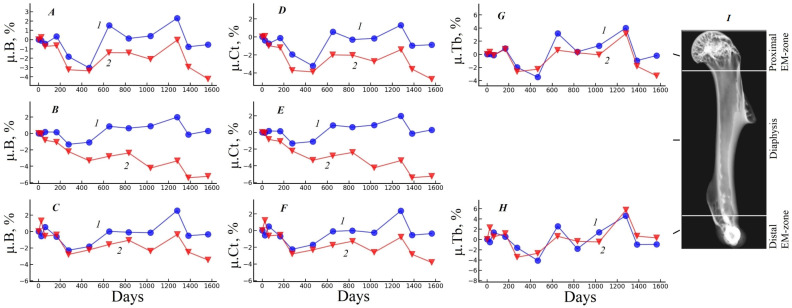
Graphs of changes in the linear attenuation coefficient of bone tissue (cortical and trabecular) μ.B (**A**–**C**); linear attenuation coefficient of cortical bone tissue μ.Ct (**D**–**F**); linear attenuation coefficient of trabecular bone tissue μ.Tb (**G**,**H**) relative to the beginning of the experiment in the main regions of the humerus (**I**). The results are expressed as percentages. Curves 1—the sample fixed in 70% ethanol; 2—the sample fixed in 10% formalin.

Graphs of the relative changes of the morphometric parameters in the basic regions of the gerbil humerus under the influence of each fixative are shown in Figure 2, Figure 3 and Figure 4. These curves are calculated as the difference between the value of the parameter on the current day of the experiment and its value on the first day of the experiment, expressed as a percentage.

Figure 2 demonstrates the plots of the relative change in total volume (TV) in the main regions of the humerus during the experiment. In all parts of the bone fixed in 70% ethanol, only a small fluctuation in total volume (TV) within the measurement error was observed. The proximal EM zone exhibited the most significant volume variation from the start to the end of the experiment, with a difference of −0.88% (Figure 2A curve 1). On the other hand, the total volume (TV) fixed in 10% formalin increased monotonically during the experiment in all parts of the bone. The maximum difference in TV was found in the distal EM-zone and was +6.11% (Figure 2C curve 2).

Similar results are observed for cortical and trabecular bone tissue in the main regions of the bone (see Figure 3). Indeed, for a sample fixed in 70% ethanol, only small fluctuations in the volume parameters of cortical (Ct.BV) and trabecular (Tb.BV) bone tissue are observed during the experiment, not exceeding the measurement error, with a maximum change of −1.71% for Ct.BV in the proximal EM-zone (see Figure 3D curve 1) and −1.91% for Tb.BV in the distal EM zone (see Figure 3H curve 1) at the end of the experiment. At the same time, the cortical (Ct.BV) and trabecular (Tb.BV) volume parameters show an increase during the experiment in all parts of the bone, reaching maximum differences at the end of the experiment of +8.63% for Ct.BV in the distal EM-zone (see Figure 3F curve 2) and +7.80% for Tb.BV in the distal EM zone (see Figure 3H curve 2), under the effect of 10% formalin.

Figure 4 illustrates the dynamics of the linear attenuation coefficients in the bone tissue of the basic regions of the humerus. For a sample fixed in 70% ethanol, the value of the absorption in cortical bone (μ.Ct) varies within the measurement error and shows a maximum difference of −0.88% in the proximal EM zone (see Figure 4D curve 1). At the same time, for the sample fixed in 10% formalin, the cortical bone absorption (μ.Ct) decreases monotonically during the experiment, reaching a maximum difference of −5.26% in the diaphysis at the end of the experiment (see Figure 4E, curve 2). The value of the linear attenuation coefficient in trabecular bone tissue (μ.Tb) exhibits variations that fall within the measurement error throughout the experiment for both fixatives (see Figure 4G,H, curves 1 and 2). The maximum relative change observed was −3.29% in the proximal EM zone for a sample preserved in 10% formalin at the conclusion of the experiment.

Following the analysis of the gerbil humerus morphometric parameters mentioned above, we can draw the following conclusions:

1. Volumetric parameters of bone.

Formalin-fixed specimen: During the experimental period, all basic regions of the humerus showed a consistent tendency to increase in volumetric parameters. Specifically, total volume (TV), bone volume (BV), cortical bone volume (Ct.BV) and trabecular bone volume (Tb.BV) showed progressive increases. The maximum increase of +8.63% was recorded for the cortical bone volume (Ct.BV) in the distal EM-zone by the end of the experiment.

Ethanol-fixed sample: During the experimental period, there were no changes exceeding the experimental spread in the volumetric parameters—total volume (TV), bone volume (BV), cortical bone volume (Ct.BV) and trabecular bone volume (Tb.BV)—in all regions of the humerus.

2. The linear attenuation coefficient of cortical bone tissue (μ.Ct).

Formalin-fixed sample shows a tendency to decrease μ.Ct during the experiment in all basic regions of the humerus. At the end of the experimental period, the maximum reduction in cortical bone tissue (μ.Ct) was observed in the diaphyseal region, with a measured decrease of −5.26%. In the ethanol-fixed specimen, no changes in the linear attenuation coefficient of cortical bone tissue (μ.Ct) exceeding the experimental spread were observed in any of the basic regions of the humerus.

3. Linear attenuation coefficient of trabecular bone tissue (μ.Tb), taking into account the spread of experimental values, remains unchanged throughout the experimental period in both the proximal and distal EM zones of the humerus. This observation was consistent for specimens fixed in both formalin and ethanol solutions.

## 4. Discussion

This study investigated the effects of long-term chemical preservation on bone tissues in two model species: thick-toed gecko and Mongolian gerbil. The gecko left forelimb third digits originated from the delayed vivarium control group of the Bion-M No. 1 orbital mission, which was conducted in 2013. This provided a unique opportunity to evaluate the outcomes of preservation following experimental protocols related to spaceflight. In parallel, we examined the humeral bone tissue of the rodent species Mongolian gerbil, which is a valuable model in gravitational biology and biomedical research.

Thick-toed geckos are not a common model organism in either terrestrial or space biology. It was first proposed as a model for a space experiment by Makarov A.N. (Institute of Human Morphology, Moscow, Russia) [20]. Unlike more extensively studied species such as the North American green anole (*Anolis carolinensis*) [21,22,23], their smaller size (15–16 cm vs. 25 cm) and presence of subdigital lamellae, which aid in adhesion under microgravity, make thick-toed geckos a promising model species for orbital studies [20,24,25]. Nevertheless, breeding them in sufficient numbers and standardizing experimental groups for flight, delayed synchronous control, and delayed vivarium control remains challenging. This was particularly a problem in 2013 during preparations for the 30-day orbital experiment aboard the Bion-M No. 1 biosatellite. As a result, there was significant weight variation within the vivarium control group, as priority was given to standardizing weights for the flight and delayed synchronisation control groups. Gecko N°21 was 24.3% heavier than gecko N°18. The snout-vent length and 4th phalanx length were also greater in gecko N°21 by 4.6% and 3.8%, respectively. Gecko N°21 likely had larger fat reserves, which could have been influenced by foraging behavior and social status.

The study of the 4th phalanx of thick-toed gecko left forelimb third digit employed high-resolution XPCT imaging to evaluate the effects of long-term storage in different fixatives on bone tissue. Interestingly, BV/TV was 5.3% higher in the smaller and lighter gecko (N°18) stored in 10% formalin. This allows us to hypothesize that fixative-induced swelling perhaps exceeded individual variability, while ethanol storage may have contributed to sample shrinkage and greater volumetric contrast.

Mongolian gerbils may be used as a valuable alternative to traditional mouse and rat models in microgravity research. Features of their physiology and behavior, as well as some similarities with humans, make them particularly useful for terrestrial and spaceflight studies [25]. Our study on Mongolian gerbil humeral bone included a quantitative assessment of bone structural parameters via micro-CT and measurement of the pH levels of the fixatives at the start and end of the preservation period.

During the experiment, no changes in the pH of the fixatives were detected, which allows us to assume their stability or insufficient sensitivity of the selected pH control method. On the other hand, our findings via micro-CT reveal substantial differences in the effects of the fixatives on gerbil bone morphometric parameters and linear attenuation coefficients.

Formalin fixation resulted in a consistent increase in volumetric morphometric parameters both cortical and trabecular bone across all regions of gerbil humerus. The most pronounced effect was observed in the cortical bone volume (Ct.BV) of the distal EM zone, which showed a maximum increase of 8.63% by the end of the experiment. This volumetric expansion may be related to the ability of formalin to induce tissue-swelling.

Bahr et al. [26] showed that tissue swelling during formalin fixation is driven primarily by the aqueous medium, with osmolarity being a critical determinant. As formaldehyde-induced cross-linking alters the native collagen architecture and formaldehyde gradually oxidizes to formic acid via the Cannizzaro reaction, mineral dissolution may progressively weaken the collagen–mineral interface, releasing the constraint on matrix expansion. This creates a coupled mechanism: demineralization, reflected in the decreasing linear attenuation coefficient, loosens the mineralized framework, while the organic matrix simultaneously swells through water uptake.

Thus, the observed increase in bone volume and decrease in μ are likely influenced by structural changes in the organic matrix, particularly collagen cross-linking and hydration, rather than swelling of the mineral phase itself, which is rigid and not prone to volumetric expansion [27,28,29].

The differential effects observed between cortical and trabecular bone may relate to differences in diffusion kinetics, surface-to-volume ratio, and mineral density. In contrast, ethanol-fixed specimens displayed remarkable stability in both volumetric and densitometric parameters, likely due to the dehydrating properties of ethanol that prevent collagen hydration and the absence of acid-generating degradation pathways. These observations underscore that the choice of fixative affects both the organic and mineral phases of bone, and that the resulting changes reflect complex interactions between fixative chemistry, osmolarity, and bone microarchitecture.

Short-term formalin fixation does not significantly alter mineral content, suggesting that mineral leaching alone cannot account for volumetric changes [27]. The interplay between mineral and organic phases at the nanoscale further indicates that modifications in the collagen network can affect tissue deformability and apparent ‘loosening’ without substantial mineral loss [30].

The observed increase in bone volume may have implications for studies relying on precise volumetric measurements, particularly in the context of space biology research where subtle changes in bone microarchitecture are of interest.

Conversely, the ethanol-fixed specimens demonstrated remarkable stability in volumetric parameters throughout the experimental period. This stability was consistent across total volume (TV), bone volume (BV), cortical bone volume (Ct.BV), and trabecular bone volume (Tb.BV) in all regions of the humerus.

Regarding the linear attenuation coefficient, our results indicate a divergence between the two fixatives. Formalin-fixed samples exhibited a decreasing trend in the linear attenuation coefficient of cortical bone tissue (μ.Ct) across all regions of the humerus. The maximum reduction—5.26% was observed in the diaphyseal region by the end of the experiment. This decrease in attenuation coefficient could be indicative of mineral loss or changes in bone density, aligning with Ref. [7], which reported continuous mineral loss in formalin-fixed rabbit tibiae.

In contrast, ethanol-fixed specimens showed no changes in the linear attenuation coefficient of cortical bone tissue (μ.Ct) in any region of the humerus exceeding the experimental variation. The preservation of original bone parameters in ethanol-fixed samples and the stability in X-ray absorption properties suggests that ethanol fixation may better preserve the mineral content of bone tissue over extended periods, which could be advantageous for studies focusing on bone mineralization or density.

Interestingly, the linear attenuation coefficient of trabecular bone tissue (μ.Tb) remained unchanged taking into account the experimental spread throughout the experimental period in both proximal and distal EM zones, regardless of the fixative used. This finding suggests that trabecular bone may be less susceptible to fixative-induced changes in mineral content or density compared to cortical bone.

The different effects of formalin and ethanol fixation observed in this study have important implications for space biology research and other fields relying on long-term preservation of bone specimens. The choice of fixative can significantly impact the interpretation of experimental findings, particularly in studies investigating microgravity-induced changes in bone parameters. For instance, in space biology experiments where accurate measurements of bone volume and density are critical, long-term storage of specimens in formalin may introduce artifacts that could be mistaken for microgravity exposure. The volumetric expansion and decrease in cortical bone attenuation observed in specimens stored in formalin could potentially mask or exaggerate the effects of microgravity on bone tissue. On the other hand, the stability demonstrated by specimens stored in ethanol suggests that this fixative may be more suitable for preserving the original bone properties in space-flight samples. These findings underscore the importance of standardized preservation protocols, particularly in the context of space experiments. The complex interactions between bone tissue and preservative solutions highlight the need for careful consideration of fixation methods when designing experiments and interpreting results [8,9,10,11,26,27,28,29,30,31,32,33].

Furthermore, our study contributes to the ongoing discussion about the effects of different preservation methods on bone properties. While some previous studies, such as Refs. [10,11], reported no significant changes in bone properties after short-term formalin fixation, our long-term study reveals notable alterations in both volumetric and X-ray absorption properties. Previously, other authors have shown the effect of long-term fixation with formalin on the mechanical properties of bone [5,6,8,27]. These data emphasize the importance of considering preservation duration when evaluating the effects of fixatives on bone tissue.

The stability observed in ethanol-fixed specimens aligns with some previous findings, such as those reported by Vesper et al. [33], who suggested that ethanol preservation may have less influence on bone mechanical properties compared to other fixatives. However, it is important to note that our study focused on morphometric and X-ray absorption properties, and further research may be needed to fully elucidate the effects of long-term ethanol fixation on these bone parameters. In future studies, it will be essential to validate these findings using a larger sample size and a standardized protocol. Caution is warranted in interpreting the gecko phalanx data presented here, as they were acquired using a different imaging modality and are therefore not directly comparable with the gerbil results; however, no inconsistencies were observed between the two data sets.

The emergence of advanced imaging techniques, particularly micro-CT, offers new opportunities for assessing fixative-induced changes in bone tissue structure and composition. Feichtinger et al. demonstrated the utility of this technique in comparing the effects of different fixatives on bone parameters [10]. The ability to non-destructively analyze bone microstructure and mineral content provides researchers with powerful tools for optimizing preservation protocols and understanding the impact of different fixatives on bone properties.

Accordingly, our study provides insight into the potential effects of long-term formalin and ethanol fixation on Mongolian gerbil humeral bone. The different impacts of these fixatives on bone volumetric and X-ray absorption properties highlight the critical need for careful selection of preservation methods in space biology research and other fields relying on long-term bone specimen storage. Future studies should consider exploring alternative fixatives or preservation techniques that can maintain both the structural and compositional integrity of bone samples over extended periods. Additionally, the integration of advanced imaging techniques like micro-CT with molecular and cellular analyses may provide a more comprehensive understanding of the effects of preservation methods on bone tissue at multiple scales.

Our findings contribute to the optimization of preservation protocols for long-term storage of bone specimens, ensuring the validity and reproducibility of future research in different fields ranging from space biology to forensic science. By enhancing our understanding of fixative-induced changes in bone properties, we can improve the accuracy of data interpretation in studies investigating bone loss in microgravity and its implications for long-term space exploration.

## 5. Conclusions

XPCT of the forelimb 4th phalanx of thick-toed gecko from the delayed vivarium control group of the 30-day Bion-M No. 1 orbital mission showed that long-term storage in 10% neutral buffered formalin (2344 days) resulted in a 5.3% increase in bone volume fraction (BV/TV) compared to a sample stored in 70% ethanol for 2309 days after an initial 35-day formalin fixation.

Micro-CT analysis of gerbil humerus confirmed that the effects of long-term preservation in formalin were regionally heterogeneous, correlating with the intrinsic microarchitectural parameters of the respective bone regions (maximal changes were revealed in cortical bone: +8.63% increase of the cortical bone volume (Ct.BV) in the distal EM-zone and −5.26% reduction of linear attenuation coefficient (μ.Ct) in the diaphyseal region). An increase in volume was also shown for trabecular bone (Tb.BV) in the proximal and distal EM zones (+6.25% and +7.8%, respectively). Prolonged formalin fixation produced also a reduction in the linear attenuation coefficient of cortical bone, particularly in the diaphyseal region. In contrast, ethanol fixation preserved both volumetric and densitometric properties of bone over time.

These results address a critical gap in our understanding of biomaterial preservation, with particular relevance to valuable samples obtained from space missions.

By revealing the different effects of formalin and ethanol fixation on bone properties, this study contributes to the optimization of preservation protocols for long-term storage of bone specimens. This knowledge is essential to ensure the validity and reproducibility of future research across a range of disciplines, including space biology, medicine and forensic science.

Future research should focus on extending this investigation to other skeletal elements and species, as well as exploring alternative fixatives and storage conditions. In addition, studies examining the effects of these fixatives over extended periods of time could provide valuable insights into the long-term stability of preserved bone samples.

## Figures and Tables

**Table 1:** Morphometric parameters of thick-toed geckos and the 4th phalanges of their left forelimb 3rd digits.

Gecko Number	Fixation and Storage	Body Mass, g	SVL, mm	Phalangeal Length, mm	Phalangeal BV, mm^3^	Phalangeal TV, mm^3^	Phalangeal BV/TV, %
N°18	10% formalin (2344 days)	14.33	71.8	0.743	0.01076	0.01365	78.8
N°21	70% ethanol (2309 days) after 35 days in 10% formalin	18.94	75.3	0.772	0.00916	0.01225	74.8
Differences		−24.3%	−4.6%	−3.8%	+17.5%	+11.4%	+5.3%

**Table 2:** Morphometric parameters of Mongolian gerbil humeral bone at the beginning and at the end of the experiment.

Parameter	70% Ethanol			10% Formalin		
	First Day	Last Day	Difference, %	First Day	Last Day	Difference, %
Whole sample						
TV, mm^3^	51.10	50.75	−0.68	50.98	52.88	3.72
BV, mm^3^	28.96	28.67	−1.01	29.21	31.11	6.51
Ct.BV, mm^3^	25.91	25.66	−0.96	26.09	27.80	6.53
Tb.BV, mm^3^	3.05	3.01	−1.42	3.12	3.31	6.31
μ.B, mm^−1^	1.162	1.155	−0.55	1.149	1.101	−4.20
μ.Ct, mm^−1^	1.196	1.191	−0.46	1.186	1.131	−4.60
μ.Tb, mm^−1^	0.873	0.870	−0.31	0.863	0.838	−2.89
Proximal EM-zone						
TV, mm^3^	20.42	20.24	−0.88	20.19	20.59	2.00
BV, mm^3^	7.54	7.42	−1.59	7.57	8.05	6.29
Ct.BV, mm^3^	4.96	4.88	−1.71	4.94	5.26	6.31
Tb.BV, mm^3^	2.57	2.54	−1.35	2.63	2.79	6.25
μ.B, mm^−1^	0.957	0.951	−0.56	0.949	0.908	−4.25
μ.Ct, mm^−1^	1.011	1.002	−0.88	1.004	0.956	−4.70
μ.Tb, mm^−1^	0.860	0.858	−0.20	0.852	0.824	−3.29
Diaphysis						
TV, mm^3^	23.71	23.54	−0.71	23.95	25.02	4.48
BV, mm^3^	16.17	16.00	−1.08	16.56	17.55	5.98
Ct.BV, mm^3^	16.11	15.93	−1.08	16.50	17.49	6.01
Tb.BV, mm^3^	-	-	-	-	-	-
μ.B, mm^−1^	1.288	1.291	0.30	1.287	1.220	−5.25
μ.Ct, mm^−1^	1.288	1.292	0.30	1.288	1.220	−5.26
μ.Tb, mm^−1^	-	-	-	-	-	-
Distal EM-zone						
TV, mm^3^	6.973	6.974	0.02	6.84	7.26	6.11
BV, mm^3^	5.250	5.252	0.03	5.08	5.52	8.56
Ct.BV, mm^3^	4.84	4.85	−0.20	4.65	5.05	8.63
Tb.BV, mm^3^	0.41	0.40	−1.91	0.43	0.47	7.80
μ.B, mm^−1^	1.096	1.092	−0.34	1.067	1.030	−3.48
μ.Ct, mm^−1^	1.106	1.102	−0.37	1.079	1.037	−3.83
μ.Tb, mm^−1^	0.956	0.946	−0.98	0.928	0.930	0.29

## Data Availability

Dataset available on request from the authors.

## Abbreviations

The following abbreviations are used in this manuscript:

micro-CT	X-ray microtomography
XPCT	X-ray phase contrast tomography
SVL	Snout-vent length (Snout–vent length (SVL) is a morphometric measurement taken in herpetology from the tip of the snout to the anterior edge of the cloacal slit)
TV	Total volume
BV	Bone volume
BV/TV	bone volume fraction
Ct.BV	Cortical bone volume
Tb.BV	Trabecular bone volume
μ.B	Linear attenuation coefficient of bone
μ.Ct	Linear attenuation coefficient of cortical bone
μ.Tb	Linear attenuation coefficient of trabecular bone
EM	Epiphysis and metaphysis
